# Diagnostic Accuracy of the Xpert MTB/RIF Assay for Lymph Node Tuberculosis: A Systematic Review and Meta-Analysis

**DOI:** 10.1155/2019/4878240

**Published:** 2019-05-19

**Authors:** Guocan Yu, Fangming Zhong, Bo Ye, Xudong Xu, Da Chen, Yanqin Shen

**Affiliations:** ^1^Department of Thoracic Surgery, Hangzhou Red Cross Hospital, Hangzhou, Zhejiang, China; ^2^Department of Tuberculosis, Hangzhou Red Cross Hospital, Hangzhou, Zhejiang, China

## Abstract

**Objectives:**

To evaluate the performance of Xpert MTB/RIF for lymph node tuberculosis (LNTB).

**Methods:**

We searched databases for published reports. We reviewed the studies and identified the performance of Xpert MTB/RIF with respect to a composite reference standard (CRS) and culture. We used a bivariate random-effects model to perform meta-analyses and used metaregression to analyze sources of heterogeneity.

**Results:**

15 independent studies compared Xpert MTB/RIF with CRS while 21 comparing it with culture were included. The pooled sensitivity and specificity of Xpert MTB/RIF were 79% and 98% compared to that of CRS, respectively, and 84% and 91% compared to that of culture, respectively. The pooled sensitivity and specificity using fine needle aspiration (FNA) samples versus CRS were 80% and 96%, whereas those against culture were 90% and 89%, respectively. The percentages while working with tissue samples versus CRS were 76% and 100%, respectively, whereas those against culture were 76% and 92%, respectively. There was no significant difference in diagnostic efficiency among the types of specimen.

**Conclusions:**

Xpert MTB/RIF demonstrates good diagnostic efficiency for LNTB and is not related to the type of specimen, obtained via different routes.

## 1. Introduction

Tuberculosis is one of the most serious challenges to global public health [1]. Besides causing pulmonary tuberculosis (PTB),* Mycobacterium tuberculosis* infection may spread to extrapulmonary sites, causing extrapulmonary tuberculosis (EPTB), LNTB being its most common type. At present, fine needle aspiration (FNA) of lymph node and biopsy are used for diagnosis, although the accuracy is relatively low, and traditional diagnostic protocols, such as* Mycobacterium tuberculosis* culture, are quite time-consuming. Mostly, biopsy with histopathological examination and culture are required for proper diagnosis; effective control of tuberculosis lies in its rapid diagnosis and treatment. Therefore, a rapid laboratory diagnosis of tuberculosis is an urgent necessity. The Xpert MTB/RIF assay is a rapid, automated molecular test with high accuracy in PTB and EPTB detection [2]. This assay has also been recommended for the diagnosis of LNTB and has shown good diagnostic efficiency [3]. However, the diagnostic efficiency with different types of lymph node specimens (FNA and tissue samples) remains controversial. Due to lack of independent systematic research of the diagnostic accuracy of Xpert MTB/RIF assay for LNTB, the possibility of influence of type of specimen (FNA and tissue samples) on the results is yet to be clarified. For this purpose, we performed a meta-analysis to confirm the diagnostic performance of Xpert MTB/RIF assay, compared to that of CRS and mycobacterial culture, in the detection of LNTB, using different types of specimen, obtained in different ways from individuals with suspected LNTB. We analyzed the pooled sensitivity and specificity of this assay against different references. Moreover, diagnostic efficiency of the test was evaluated, based on different types and conditions of samples, by subgroup analysis.

## 2. Methods

### 2.1. Data Sources and Search Strategy

On Jul 6, 2018, we searched PubMed, Embase, the Cochrane Library, China National Knowledge Infrastructure (CNKI), and the Wanfang database for studies evaluating the accuracy of Xpert in LNTB detection. The search formula ((Xpert OR Gene Xpert) AND (Tuberculosis, Lymph Node”[Mesh] OR ”Extra pulmonary tuberculosis”)) was used for PubMed without any limitation. Similar search formulae were used for Embase, the Cochrane Library, CNKI, and Wanfang databases. References cited in the included articles and reviews were further explored for possible candidate studies.

### 2.2. Inclusion Criteria

We included full-text original studies that assessed the diagnostic accuracy of Xpert assay for LNTB using FNA or biopsy tissue specimens. Reference standards were well-defined and appropriate in the studies. The articles directly provided true positive (TP), false positive (FP), false negative (FN), and true negative (TN) values for the assay or included the data necessary to calculate these measures. Case reports, studies with < 10 samples, conference reports, and abstracts without full articles were excluded.

### 2.3. Reference Standard

A composite reference standard (CRS) or mycobacterial culture was defined as the reference standard in our study. Clinical manifestation, biochemical test results, histopathology, smears, other nucleic acid amplification tests (NAATs), culture, and response to antituberculosis treatment constituted the reference standards in CRS.

### 2.4. Literature Screening and Selection

Two investigators independently assessed the candidate articles by reviewing titles and abstracts, followed by the full text, for inclusion. Discrepancies between the two investigators were resolved by discussion with a third investigator.

### 2.5. Data Extraction

We extracted data including author, year, country, TP, FP, FN, and TN values for the assay, reference standard, and specimen type, along with other parameters. The same two investigators independently extracted the necessary information from each of the included articles; we cross-checked the information obtained by them. Discrepancies between the two datasets were settled by discussion with a third investigator, similar to that during the literature selection phase. Data from studies against two different reference standards were treated separately.

### 2.6. Assessment of Study Quality

According to the two reference standards (CRS and culture), the two investigators independently divided the studies into two groups and used a revised tool for Quality Assessment of Diagnostic Accuracy Studies (QUADAS-2) to assess study quality separately [4]. Publication bias was not assessed, since these methods were not applicable to studies of diagnostic accuracy [5].

### 2.7. Data Synthesis and Statistical Analysis

We first obtained the values corresponding to TP, FP, FN, and TN in each included study and calculated the estimated pooled sensitivity and specificity of Xpert MTB/RIF associated with 95% CI, against CRS or culture, using bivariate random-effects models. Forest plots for sensitivity and specificity were generated for each study. The area under summary receiver operating characteristic (SROC) curves (AUC) was subsequently calculated. I^2^ statistics was used to assess heterogeneity between the studies and a reference standard. While 0% indicated no observed heterogeneity, values greater than 50% were considered to signify substantial heterogeneity [6, 7]. We explored different types of samples, decontamination method, sample conditions, and homogenization as potential sources of heterogeneity, using subgroup and metaregression analyses. At least four published studies were required to carry out the meta-analysis for a predefined variable type. Data from studies against CRS and culture were analyzed separately. Stata version 14.0 (Stata Corp, College Station, TX) with the midas command packages was used to generate forest plots of sensitivity and specificity with 95% CI for each study and carry out meta-analyses and metaregression analyses.

### 2.8. Imperfect Reference Standard

Imperfect reference standards may lead to misclassification of samples in diagnostic validity studies [8, 9]. For the paucibacillary nature of EPTB, a culture would be an imperfect reference standard and lead to an underestimation of the true specificity of Xpert MTB/RIF. A CRS is a composite standard that comprises results from several tests; however, a CRS itself may have reduced specificity, thereby leading to apparent FN Xpert MTB/RIF results, an underestimation of the true sensitivity of Xpert MTB/RIF [9, 10]. Therefore, a study comparing Xpert MTB/RIF with both culture and CRS might provide a more credible range for sensitivity and specificity.

## 3. Results

### 3.1. Identification of Studies and Study Characteristics

**Figure 1:**
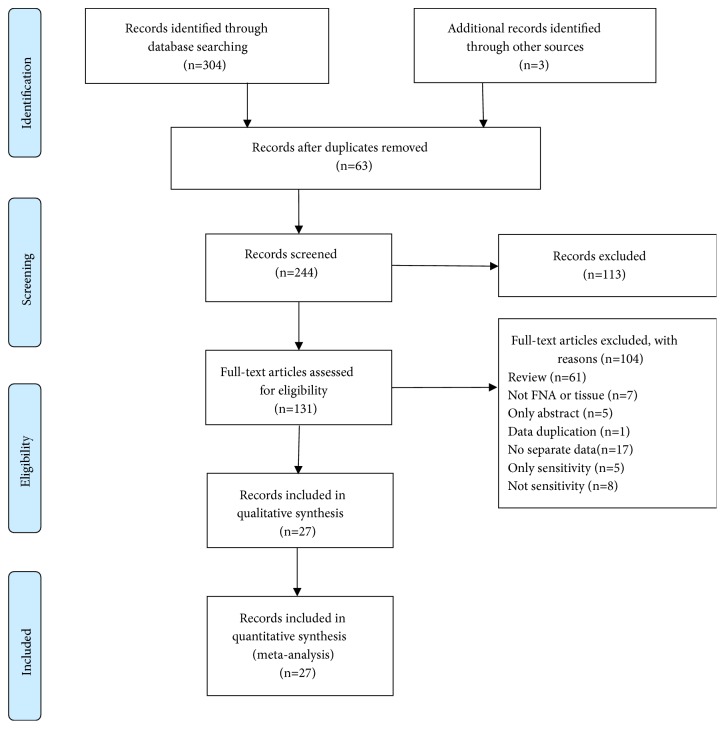
Literature retrieval flow chart. 80, 23, 159, 30, and 12 articles were found from PubMed, the Cochrane Library, Embase, Wanfang database, and CNKI respectively.

Three hundred and four candidate articles, identified from relevant databases using our search strategy, three articles identified from other sources, and twenty-seven qualified articles were included according to the inclusion criteria (Figure 1) [11–37]. The number of specimens evaluated in each article ranged from 11 to 348 with a median of 118. Twenty-four articles were written in English and three in Chinese. We excluded one study that had the same data as another included study [38] and five other articles that reported sensitivity only, without reporting any specificity [39–43].

When an article reported the use of two different standards in the same study, we considered the article to include two independent studies. In accordance with this principle, 36 independent studies were included: 15 compared Xpert MTB/RIF with CRS and 21 compared Xpert MTB/RIF with culture (Table 1). Twenty-one studies used FNA samples and 15 used biopsy tissue samples.

### 3.2. Study Quality

**Figure 2:**
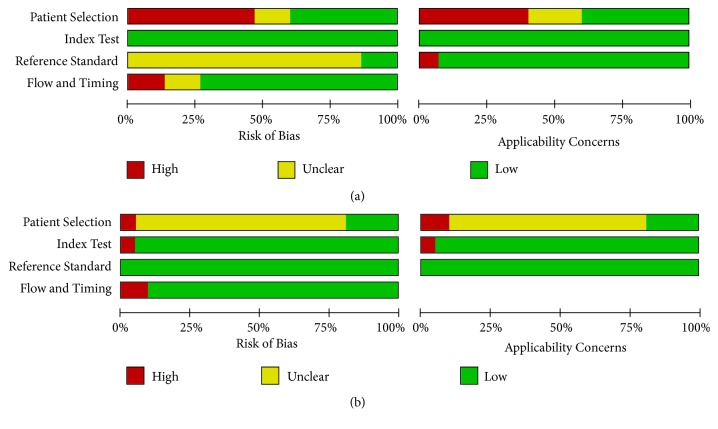
Methodological quality graphs (risk of bias and applicability concerns) as percentages across the included studies. (a) Composite reference standard. (b) Culture reference standard.

The overall methodological quality of the included studies, using a CRS and culture, is summarized in Figure 2.

### 3.3. Diagnostic Accuracy of Xpert MTB/RIF Assay for LNTB Detection

**Figure 3:**
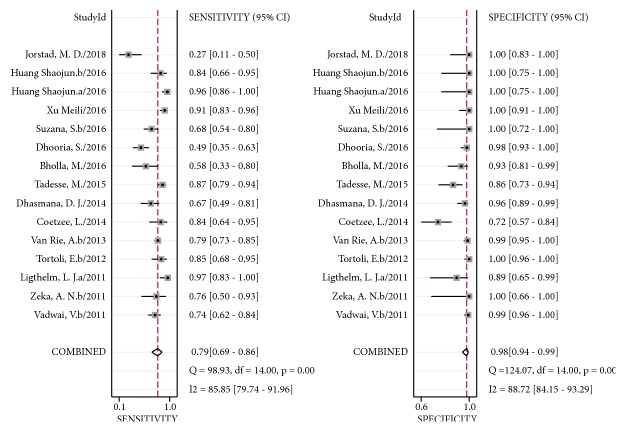
Forest plot of Xpert sensitivity and specificity for tuberculosis detection in LNTB compared with a composite reference standard. The squares represent the sensitivity and specificity of a study, and the black line represents their confidence intervals. The diamonds represent the pooled sensitivity and specificity and their confidence intervals. LNTB: lymph nodes tuberculosis.

Fifteen studies included comparison of 1597 FNA or tissue samples with a CRS; Xpert MTB/RIF sensitivity ranged from 49% (95% CI 35–63%) to 97% (95% CI 83–100%). Pooled sensitivity of Xpert MTB/RIF assay for LNTB was 79% (95% CI 69–86%) and I^2^ statistical values were 86%. Xpert MTB/RIF specificity ranged from 72% (95% CI 57–84%) to 100% (95% CI 97–100%). Pooled specificity of Xpert MTB/RIF assay was 98% (95% CI 94–99%) and I^2^ statistical values were 89% (Figure 3). The heterogeneity of sensitivity and specificity was significant. The AUC of SROC was 0.96 (95% CI 0.94–0.98).

**Figure 4:**
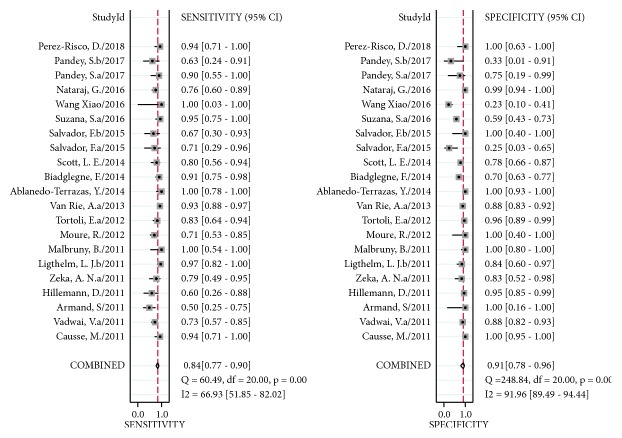
Forest plot of Xpert sensitivity and specificity for tuberculosis detection in LNTB compared with culture reference standard. The squares represent the sensitivity and specificity of a study, and the black line represents their confidence intervals. The diamonds represent the pooled sensitivity and specificity and their confidence intervals. LNTB: lymph nodes tuberculosis.

Compared to a culture reference standard, the pooled sensitivity of Xpert MTB/RIF was 84% (95% CI 77–90%) with I^2^ = 67% and specificity was 91% (95% CI 78–96%) with I^2^ = 92% for 1629 FNA or tissue specimens from 21 studies (Figure 4). The heterogeneity of sensitivity was acceptable; however, that of specificity was significant. The AUC of SROC was 0.92 (95% CI 0.89–0.94) versus that of culture, suggesting excellent overall diagnostic validity.

We explored the heterogeneity among studies, using subgroup and metaregression analyses on predefined subgroups of sample types, decontamination method, sample condition, and homogenization used in the assay.

**Figure 5:**
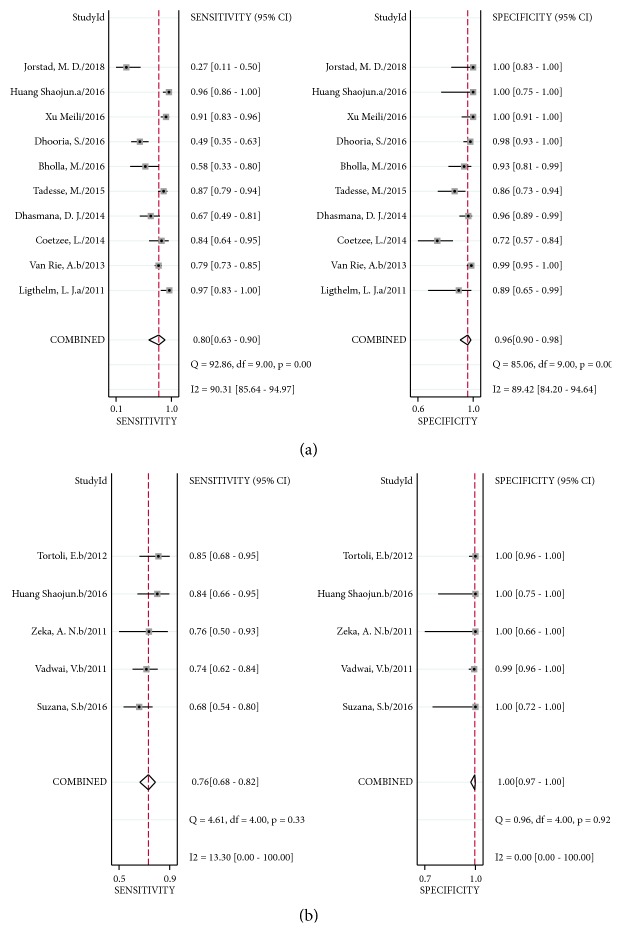
Forest plot of Xpert sensitivity and specificity for tuberculosis detection in LNTB versus composite reference standard. (a) FNA samples. (b) Tissue samples. The squares represent the sensitivity and specificity of a study, and the black line represents their confidence intervals. The diamonds represent the pooled sensitivity and specificity and their confidence intervals. LNTB: lymph nodes tuberculosis. FNA: fine needle aspiration.

In case of Xpert MTB/RIF using FNA samples, sensitivity ranged from 27% (95% CI 11–50%) to 97% (95% CI 83–100%) against a CRS. The pooled sensitivity was 80% (95% CI 63–90%) with I^2^ = 90%, and the pooled specificity was 96% (95% CI 90–98%) with I^2^ = 89% (Figure 5(a)). There was significant heterogeneity in sensitivity and specificity among studies of Xpert MTB/RIF assay using FNA samples compared to a CRS. The AUC of SROC was 0.96 (95% CI 0.94–0.97), suggesting very good overall diagnostic validity. When using tissue samples, sensitivity ranged from 68% (95% CI 54–80%) to 85% (95% CI 68–95%), and specificity ranged from 99% (95% CI 96–10%) to 100% (95% CI 96–100%) against a CRS. The pooled sensitivity and specificity of Xpert MTB/RIF assay using tissue samples versus CRS were 76% (95% CI 68–82%) with I2 = 13%, and 100% (95% CI 97–100%) with I2 = 0 (Figure 5(b)). Heterogeneity across studies of Xpert MTB/RIF assay using tissue samples compared to that of CRS was not obvious. The AUC of SROC was 0.96 (95% CI 0.94–0.98).

**Figure 6:**
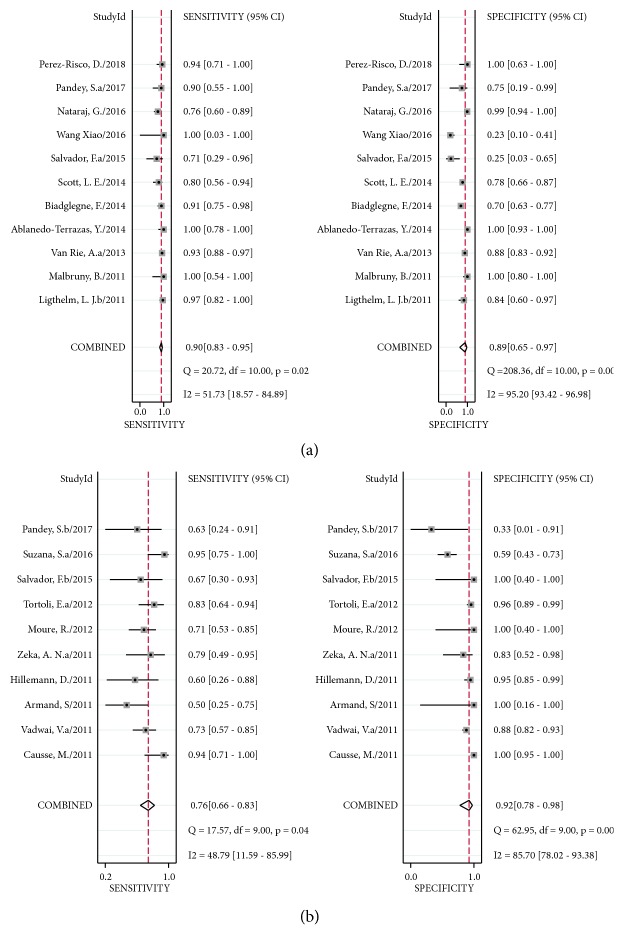
Forest plot of Xpert sensitivity and specificity for tuberculosis detection in LNTB versus culture. (a) FNA samples. (b) Tissue samples. The squares represent the sensitivity and specificity of a study, and the black line represents their confidence intervals. The diamonds represent the pooled sensitivity and specificity and their confidence intervals. LNTB: lymph nodes tuberculosis. FNA: fine needle aspiration.

In Xpert MTB/RIF using FNA samples, sensitivity ranged from 71% (95% CI 29–96%) to 100% (95% CI 78–100%) against culture. The pooled sensitivity was 90% (95% CI 83–95%) with I^2^ = 52%, and pooled specificity was 89% (95% CI 65–97%) with I^2^ = 95% (Figure 6(a)). While heterogeneity of sensitivity was acceptable, there was significant heterogeneity of specificity when using FNA samples, compared to that with culture. The AUC of SROC was 0.94 (95% CI 0.92–0.96). When using tissue samples, sensitivity ranged from 50% (95% CI 25–75%) to 95% (95% CI 75–100%), and specificity ranged from 33% (95% CI 1–91%) to 100% (95% CI 95–100%) against culture. The pooled sensitivity and specificity of Xpert MTB/RIF assay using tissue samples versus culture were 76% (95% CI 66–83%) with I^2^ = 49%, and 92% (95% CI 78–98%) with I^2^ = 86% (Figure 6(b)). Heterogeneity was very obvious with tissue samples compared to that with culture. The AUC of SROC was 0.86 (95% CI 0.83–0.89).

Metaregression analysis showed that studies with FNA samples and tissue samples, compared to a CRS, showed similar sensitivity (79% and 78%, metaregression P = 0.35). However, studies with FNA samples, compared to a CRS, showed lower specificity (96%) than those using tissue samples (100%); the difference was statistically significant (metaregression P < 0.01). The sensitivity of studies using FNA samples was higher than that of studies using tissue samples, compared to culture (90% versus 76%); however, the difference was not statistically significant (meta-regression P = 0.43). The specificity of studies using FNA samples was lower than that of studies using tissue samples, compared to culture (89% versus 93%); this difference was also not statistically significant (metaregression P = 0.56).

Metaregression analysis showed that decontamination method (with NALC-NaOH or without), sample condition (fresh or frozen), homogenization (mechanical or otherwise), and patient population (high/low income) did not have any effect on Xpert MTB/RIF sensitivity and specificity (meta-regression P > 0.05), compared to that in CRS. These factors were, therefore, obviously not a source of heterogeneity across the studies.

## 4. Discussion

Diagnosis of EPTB, including LNTB, is very challenging, due to the characteristics of a low bacterial load. In most cases, invasive examinations are necessary. For LNTB, the most commonly used invasive procedures are FNA and biopsy. Specimens obtained by different methods have different sensitivity for the diagnosis of LNTB. Sensitivity of pathological diagnosis is the highest with the most invasive biopsy specimens [26]. FNA is widely used in the diagnosis of LNTB, due to its simplicity and safety, but its sensitivity ranges from 10% to 80% for a positive mycobacterial culture [44, 45]. The preferred method of obtaining specimens from patients suspected with LNTB remains controversial. Several published studies had reported positive Ziehl-Neelsen staining and culture to correlate to FNA and biopsy [26, 44]. On the contrary, granulomatous inflammation observed in biopsy specimens was much more frequent than in FNA specimens (96.8% versus 54.8%, respectively, P < 0.001) [26]. This finding was helpful in the diagnosis of Ziehl-Neelsen stain- and culture-negative cases.

Nucleic acid detection has been widely used in the diagnosis of tuberculosis [46]. The Xpert MTB/RIF assay is currently one of the most commonly used nucleic acid detection methods. This test can confirm rifampicin resistance while validating the MTB complex DNA within 2 h. It is widely used for diagnosing PTB and EPTB. Several reports on its diagnostic efficiency have been published [47]. The assay has also been recommended by WHO for the diagnosis of LNTB. Systematic review and meta-analysis published in 2014 and 2015 showed good diagnostic efficiency of Xpert MTB/RIF for LNTB [3, 48]. However, there has been no consensus on the sensitivity of this test for specimens (FNA and biopsy specimens) obtained by different methods. For this purpose, we designed this study to determine the performance of Xpert MTB/RIF in FNA and biopsy specimens and provide references for the selection of specimens in the diagnosis of LNTB.

Our research found that the pooled sensitivity and specificity of Xpert MTB/RIF for LNTB were 79% (95% CI 69–86%) and 98% (95% CI 94–99%) versus CRS and 84% (95% CI 77–90%) and 91% (95% CI 78–96%) versus culture, respectively. In a meta-analysis by Denkinger et al., Xpert MTB/RIF pooled sensitivity and specificity were 81.2% (95% CI 72.4–87.7%) and 99.1% (95% CI 94.5–99.9%) versus CRS, and 83.1% (95% CI 71.4–90.7%) and 93.6% (95% CI 87.9–96.8%) versus culture for lymph node tissues or aspirates [3]. The results were similar to our study; however, it failed to make further analysis based on the type of specimens. In the present study, significant heterogeneity was also observed among the studies. Subgroup analysis revealed the pooled sensitivity of Xpert MTB/RIF, performed on FNA samples, to be 80% (95% CI 63–90%) and 90% (95% CI 83–95%) and the pooled specificity to be 96% (95% CI 90–98%) and 89% (95% CI 65–97%) compared to CRS and culture, respectively. The pooled sensitivity and specificity were 76% (95% CI 68–82%) and 76% (95% CI 66–83%) and 100% (95% CI 97–100%) and 92% (95% CI 78–98%) compared to CRS and culture, when using tissue specimens. Metaregression analysis revealed that the sensitivity of Xpert MTB/RIF using FNA specimens was higher than that of using tissue specimens, regardless of the gold standard, although statistical difference was not significant. The sample reagent buffer used in the Xpert assay had originally been designed for the liquefaction of mucoid sputum samples. Tissues may not be completely homogenized with the same buffer, thus resulting in low sensitivity [30]; FNA specimens are more likely to be homogenized. This may possibly explain why FNA specimens have higher sensitivity than tissues. On the contrary, the specificity of Xpert MTB/RIF using FNA specimens was lower than that of using tissue specimens regardless of the gold standard; statistical difference was significant when considered against a CRS. Overall, the diagnostic efficiency of Xpert MTB/RIF for LNTB, using FNA and tissue specimens, was found to be similar. Owing to its paucibacillary nature, for LNTB, a CRS might be a more applicable reference standard. However, the CRS varied across studies in this research. CRS for all studies included the results of culture while that for most studies included the results of histology/cytology and smear microscopy; a few studies included the clinical features and radiology results, three studies included the response to antituberculosis treatment, and only one study included the result of Xpert MTB/RIF [19]. This might be one of the sources of heterogeneity across studies.

Sample processing of lymph node specimens, such as decontamination, sample condition, and homogenization, was variable across studies, but metaregression analysis showed that these factors did not affect the result and hence were not the sources of heterogeneity. In addition, we also found the patient's financial status to not affect the outcome.

According to the findings of this study, a multistep approach could be adopted for the management of suspected LNTB: FNA should be performed as the first step due to its less invasiveness, followed by complete relevant examinations, including Xpert MTB/RIF and pathological tests to improve diagnostic sensitivity. When the inspection result of the first step is negative, a more invasive technique (like biopsy) should be performed; pathological examination may be used for further validation, rather than Xpert MTB/RIF, since the assay did not increase the sensitivity of diagnosis when performed in biopsy-obtained samples.

Our meta-analysis also had several limitations. We realize that we may have missed some studies, despite the comprehensive search, and some studies that failed to distinguish specimen types. In addition, some included studies used multiple sample types, which may have led to some bias in our results. In addition, sample processing of lymph node specimens was highly variable across and within studies, since the assay, designed for respiratory samples, may slightly vary for other specimens. Additionally, the CRS standard for the studies was also different. Heterogeneity among the studies was remarkable, and the pooled estimates need to be interpreted with caution.

## 5. Conclusions

In this meta-analysis, we observed that the pooled sensitivity and specificity of Xpert MTB/RIF were 79% and 98%, respectively, when compared with a CRS, and 84% and 91%, respectively, when compared with culture. When performed on FNA samples, the pooled sensitivity and specificity were 80% and 96% versus CRS and 90% and 89% versus culture, respectively. When performed on tissue samples, the pooled sensitivity and specificity were 76% and 100% versus CRS, and 76% and 92% versus culture, respectively. There was no significant difference in the diagnostic efficiency for specimens obtained via different routes. Xpert MTB/RIF showed a good diagnostic efficiency on LNTB and was not related to the type of specimen.

## Figures and Tables

**Table 1:** Characteristics of the included studies. FNA: fine needle aspiration. CRS: composite reference standard. TP: true positive. FP: false positive. FN: false negative. TN: true negative.

Author	Year	County	TP	FP	FN	TN	Specimen type	Reference standard	Decontaminate method	Sample condition	Location	Homogenisation	Sample ratio	Patient selection method
Causse, M.	2011	Spain	16	0	1	70	Tissue	Cuture	NALC-NaOH	Fresh	Peripheral	No	2:1	consecutive

Vadwai, V.a	2011	India	32	17	12	127	Tissue	Cuture	No	Fresh	Peripheral	Mechanical	2:1	consecutive

Vadwai, V.b	2011	India	49	1	17	122	Tissue	CRS	No	Fresh	Peripheral	Mechanical	2:1	consecutive

Armand, S	2011	France	8	0	8	2	Tissue	Cuture	NALC-NaOH	Frozen	Peripheral	Mechanical	3:1	Convenience

Hillemann, D.	2011	Germany	6	3	4	52	Tissue	Cuture	NALC-NaOH	Fresh	Peripheral	Mechanical	3:1	Consecutive

Zeka, A. N.a	2011	Turkey	11	2	3	10	Tissue	Cuture	No	Frozen	Peripheral	No	3:1	Consecutive

Zeka, A. N.b	2011	Turkey	13	0	4	9	Tissue	CRS	No	Frozen	Peripheral	No	3:1	Consecutive

Ligthelm, L. J.a	2011	South Africa	29	2	1	16	FNA	CRS	No	Fresh	Peripheral	No	2:1	Consecutive

Ligthelm, L. J.b	2011	South Africa	28	3	1	16	FNA	Cuture	No	Fresh	Peripheral	No	2:1	Consecutive

Malbruny, B.	2011	France	6	0	0	17	FNA	Cuture	NALC-NaOH	Fresh	Peripheral	Mechanical	3:1	Convenience

Moure, R.	2012	Spain	24	0	10	4	Tissue	Cuture	NALC-NaOH	Frozen	Peripheral	Mechanical	2:1	Convenience

Tortoli, E.a	2012	Italy	24	4	5	85	Tissue	Cuture	NALC-NaOH	Frozen	Peripheral	Mechanical	2:1	Convenience

Tortoli, E.b	2012	Italy	28	0	5	85	Tissue	CRS	NALC-NaOH	Frozen	Peripheral	Mechanical	2:1	Convenience

Van Rie, A.a	2013	South Africa	139	23	10	172	FNA	Cuture	No	Fresh	Peripheral	No	2:1	Consecutive

Van Rie, A.b	2013	South Africa	160	2	42	144	FNA	CRS	No	Fresh	Peripheral	No	2:1	Consecutive

Ablanedo-Terrazas, Y.	2014	Mexico	15	0	0	53	FNA	Cuture	NALC-NaOH	Frozen	Peripheral	No	2:1	Convenience

Biadglegne, F.	2014	Ethiopia	29	56	3	132	FNA	Cuture	NALC-NaOH	Frozen	Peripheral	No	3:1	Convenience

Coetzee, L.	2014	South Africa	21	13	4	34	FNA	CRS	No	Fresh	Peripheral	No	2:1	Convenience

Dhasmana, D. J.	2014	United Kingdom	24	3	12	77	FNA	CRS	No	Fresh	Mediastinal	No	Unknow	Convenience

Scott, L. E.	2014	South Africa	16	14	4	50	FNA	Cuture	NALC-NaOH	Fresh	Peripheral	No	2:1	Convenience

Salvador, F.a	2015	Spain	5	6	2	2	FNA	Cuture	No	Fresh	Peripheral	No	Unknow	Convenience

Salvador, F.b	2015	Spain	6	0	3	4	Tissue	Cuture	No	Fresh	Peripheral	No	Unknow	Convenience

Tadesse, M.	2015	Ethiopia	76	7	11	42	FNA	CRS	NALC-NaOH	Frozen	Peripheral	Mechanical	3:1	Consecutive

Bholla, M.	2016	Tanzania	11	3	8	40	FNA	CRS	No	Fresh	Peripheral	No	3:1	Convenience

Dhooria, S.	2016	India	26	2	27	92	FNA	CRS	No	Fresh	Mediastinal	No	2:1	Convenience

Suzana, S.a	2016	India	19	19	1	27	Tissue	Cuture	No	Fresh	Peripheral	No	2:1	Consecutive

Suzana, S.b	2016	India	38	0	18	11	Tissue	CRS	No	Fresh	Peripheral	No	2:1	Consecutive

Wang Xiao	2016	China	1	24	0	7	FNA	Cuture	No	Fresh	Peripheral	No	2:1	Convenience

Nataraj, G.	2016	India	29	1	9	87	FNA	Cuture	No	Fresh	Peripheral	No	2:1	Convenience

Xu Meili	2016	China	73	0	7	40	FNA	CRS	No	Fresh	Peripheral/Mediastinal	Mechanical	2:1	Convenience

Huang Shaojun.a	2016	China	48	0	2	13	FNA	CRS	NALC-NaOH	Fresh	Peripheral	Mechanical	2:1	Convenience

Huang Shaojun.b	2016	China	26	0	5	13	Tissue	CRS	NALC-NaOH	Fresh	Peripheral	Mechanical	2:1	Convenience

Pandey, S.a	2017	Australia	9	1	1	3	FNA	Cuture	NALC-NaOH	Fresh	Peripheral	No	3:1	Convenience

Pandey, S.b	2017	Australia	5	2	3	1	Tissue	Cuture	NALC-NaOH	Fresh	Peripheral	No	3:1	Convenience

Jorstad, M. D.	2018	Zanzibar	6	0	16	20	FNA	CRS	No	Fresh	Peripheral	No	Unknow	Convenience

Perez-Risco, D.	2018	Spain	16	0	1	8	FNA	Cuture	NALC-NaOH	Frozen	Peripheral	No	2:1	Convenience

## Supplementary Materials

Supplementary Materials Search strategies for each database.
