# Supplementary material for: Diagnostic Accuracy of the Xpert MTB/RIF Assay for Lymph Node Tuberculosis: A Systematic Review and Meta-Analysis
Source: Biomed Res Int. 2019 May 19;2019:4878240. doi: 10.1155/2019/4878240 (PMC6545759; doi:10.1155/2019/4878240)
Supplement: Supplementary Materials — Search strategies for each database. [file 4878240.f1.docx]

Pubmed

#1 "Tuberculosis, Lymph Node"[Mesh] OR "Lymphadenitis, Cervical Tuberculous" OR "Tuberculous Lymphadenitis, Cervical" OR "Lymph Node Tuberculoses" OR "Lymph Node Tuberculosis" OR "Tuberculoses, Lymph Node" OR "Lymphadenitis, Tuberculous" OR "Tuberculous Lymphadenitis" OR Scrofula OR Scrofulas OR "Mycobacterial Cervical Lymphadenitis" OR "Cervical Lymphadenitis, Mycobacterial" OR "Lymphadenitis, Mycobacterial Cervical" OR "Cervical Tuberculous Lymphadenitis" OR "Extra pulmonary tuberculosis" OR " Extrapulmonary tuberculosis"

#2 xpert OR genexpert

#3 #1 and #2

Embase

#1 'tuberculous lymphadenitis'/exp OR 'Lymphadenitis, Cervical Tuberculous' OR 'Tuberculous Lymphadenitis, Cervical' OR 'Lymph Node Tuberculoses' OR 'Lymph Node Tuberculosis' OR 'Tuberculoses, Lymph Node' OR 'Lymphadenitis, Tuberculous' OR 'Tuberculous Lymphadenitis' OR Scrofula OR Scrofulas OR 'Mycobacterial Cervical Lymphadenitis' OR 'Cervical Lymphadenitis, Mycobacterial' OR 'Lymphadenitis, Mycobacterial Cervical' OR 'Cervical Tuberculous Lymphadenitis' OR 'extrapulmonary tuberculosis'/exp OR 'extra pulmonary tuberculosis'

#2 'xpert'/exp OR xpert OR genexpert

#3 #1 and #2

Cochrane

#1 "Tuberculosis, Lymph Node"[Mesh] OR "Lymphadenitis, Cervical Tuberculous" OR "Tuberculous Lymphadenitis, Cervical" OR "Lymph Node Tuberculoses" OR "Lymph Node Tuberculosis" OR "Tuberculoses, Lymph Node" OR "Lymphadenitis, Tuberculous" OR "Tuberculous Lymphadenitis" OR Scrofula OR Scrofulas OR "Mycobacterial Cervical Lymphadenitis" OR "Cervical Lymphadenitis, Mycobacterial" OR "Lymphadenitis, Mycobacterial Cervical" OR "Cervical Tuberculous Lymphadenitis" OR "Extra pulmonary tuberculosis" OR " Extrapulmonary tuberculosis"

#2 xpert OR genexpert

#3 #1 and #2

Wanfang and CNKI

#1 淋巴结结核 OR 淋巴结核 OR 结核性淋巴结炎 OR 肺外结核

#2 xpert OR genexpert

#3 #1 and #2
